# FlyNet: a versatile network prioritization server for the *Drosophila* community

**DOI:** 10.1093/nar/gkv453

**Published:** 2015-05-05

**Authors:** Junha Shin, Sunmo Yang, Eiru Kim, Chan Yeong Kim, Hongseok Shim, Ara Cho, Hyojin Kim, Sohyun Hwang, Jung Eun Shim, Insuk Lee

**Affiliations:** Department of Biotechnology, College of Life Science and Biotechnology, Yonsei University, Seoul, Korea

## Abstract

*Drosophila melanogaster* (fruit fly) has been a popular model organism in animal genetics due to the high accessibility of reverse-genetics tools. In addition, the close relationship between the *Drosophila* and human genomes rationalizes the use of *Drosophila* as an invertebrate model for human neurobiology and disease research. A platform technology for predicting candidate genes or functions would further enhance the usefulness of this long-established model organism for gene-to-phenotype mapping. Recently, the power of network prioritization for gene-to-phenotype mapping has been demonstrated in many organisms. Here we present a network prioritization server dedicated to *Drosophila* that covers ∼95% of the coding genome. This server, dubbed FlyNet, has several distinctive features, including (i) prioritization for both genes and functions; (ii) two complementary network algorithms: direct neighborhood and network diffusion; (iii) spatiotemporal-specific networks as an additional prioritization strategy for traits associated with a specific developmental stage or tissue and (iv) prioritization for human disease genes. FlyNet is expected to serve as a versatile hypothesis-generation platform for genes and functions in the study of basic animal genetics, developmental biology and human disease. FlyNet is available for free at http://www.inetbio.org/flynet.

## INTRODUCTION

*Drosophila* remains an important model organism even after a century of research. Numerous biological processes that have been evolutionary conserved across species, such as embryogenesis and canonical cell signalling pathways, have been genetically dissected in *Drosophila*. Approximately half of *Drosophila* protein sequences have mammalian homologs (1), and ∼75% of known human disease genes have *Drosophila* orthologs (2). *Drosophila* therefore is expected to be an effective model for human disease research. Indeed, several recent studies have utilized *Drosophila* to identify genes associated with human diseases and traits (3–5).

Several reverse-genetics resources are available for *Drosophila*. These resources are based on the transposon insertion mutant libraries and RNA interference (RNAi) of both cell-based and organismal systems (6–9). However, unbiased genome-wide screens are costly and suffer from high false discovery rates. Reverse-genetics screens for *Drosophila* phenotypes become more efficient through systematic gene prioritization. One increasingly popular approach for gene prioritization is network prioritization, which is based on the functional closeness to the known phenotype genes within the gene networks. The concepts underlying network prioritization as well as its efficacy for *gene-to-function* and *gene-to-phenotype* mapping have recently been reviewed (10–12).

Here we present a network prioritization server for *Drosophila* dubbed FlyNet. Although network prioritization servers exist for multiple organisms, to the best of our knowledge FlyNet represents the first network prioritization server dedicated to *Drosophila*. FlyNet is highly accurate and can effectively predict *gene-to-phenotype* associations. This *Drosophila*-specific server further benefits from using *Drosophila*-specific information, such as the gene expression atlas, across cell types and developmental stages. Such information can help users generate more reliable hypotheses relevant to the spatiotemporal context and select more confident candidates. FlyNet also can prioritize *Drosophila* genes for human diseases with an option for the integration of human genetic evidence, such as genome-wide association studies (GWAS) and *de novo* mutation screens.

## NETWORK CONSTRUCTION

The procedure that we used to construct our network consists of three major steps: (i) inferring co-functional gene pairs from various data sources; (ii) benchmarking networks inferred from individual data sources to assign likelihood scores for network links and (iii) integrating all component networks using a modified naive Bayesian approach. The FlyNet gene network was constructed based on genome build release 5.54 from FlyBase (13). A set of gold-standard functional gene pairs was generated based on the Gene Ontology Biological Process (GO-BP) (14) and MetaCyc pathway terms (15). The co-functional links in FlyNet were inferred from *Drosophila*-derived data with the following computational algorithms: co-citation (CC) of gene names among PubMed abstracts; co-expression (CX) of genes in 53 experimental series comprising 1873 microarray samples in the Gene Expression Omnibus (GEO) database (16); domain co-occurrence (DC) between proteins (17); functional links by genomic context based on gene neighborhood (GN) (18) and phylogenetic profile similarity (PG); protein–protein interactions via high-throughput assays (HT) and literature-based links (LC) from the iRefWeb meta-database version 13 (19). Additional links were inferred from published (17,20,21) and unpublished networks for other organisms. A total of 21 component networks were incorporated into FlyNet, and are summarized in Table 1 and Supplementary Table S1. These networks were benchmarked against the gold-standard gene pairs using a log-likelihood score scheme and then integrated into a single network, FlyNet, using a weighted sum method, as has been described for other published networks (17,20,21). Detailed information and methodologies are described in the Supplementary Online Methods. The integrated FlyNet gene network contains 13 119 genes (∼95% of the coding genes) and 779 484 links.

**Table 1.** Summary of the 21 component networks for FlyNet

Code	Description	# Genes	# Links
DM-CC	Co-citation of two fly genes across Medline and PubMed Central	6027	503 475
DM-CX	Co-expression of two fly genes in high-dimensional gene expression data from the GEO database (16)	11 718	275 033
DM-DC	Co-occurrence of protein domains between two fly genes (17)	4407	7604
DM-GN	Chromosomal proximity between bacterial orthologs of two fly genes in bacterial genomes (18)	1979	15 820
DM-HT	Protein–protein interactions (PPIs) identified by high-throughput assays in iRefWeb database (19)	7759	25 519
DM-LC	Protein–protein interactions (PPIs) identified by small-scale experiments collected via literature curation in iRefWeb database (19)	1202	2226
DM-PG	Phylogenetic profile similarity between two fly genes	3357	80 506
AT-CC	Orthology transfer of co-citation links in an *A. thaliana* network (21)	1747	17 501
AT-CX	Orthology transfer of co-expression links in an *A. thaliana* network (21)	1105	9455
AT-HT	Orthology transfer of high-throughput PPI in an *A. thaliana* network (21)	1013	2823
AT-LC	Orthology transfer of literature curated PPI in an *A. thaliana* network (21)	856	1977
CE-CX	Orthology transfer of co-expression links in a *C. elegans* network (20)	1434	17 497
DR-CX	Orthology transfer of co-expression links in a *D. rerio* network	3223	55 515
HS-CX	Orthology transfer of co-expression links in a *H. sapiens* network	3366	32 482
HS-HT	Orthology transfer of high-throughput PPI in a *H. sapiens* network	2741	12 520
HS-LC	Orthology transfer of literature curated PPI in a *H. sapiens* network	5254	50 488
SC-CC	Orthology transfer of co-citation links in a *S. cerevisiae* network (17)	2449	48 473
SC-CX	Orthology transfer of co-expression links in a *S. cerevisiae* network (17)	1674	18 488
SC-GT	Orthology transfer of genetic interactions in a *S. cerevisiae* network (17)	1254	6482
SC-HT	Orthology transfer of high-throughput PPI in a *S. cerevisiae* network (17)	1622	18 300
SC-LC	Orthology transfer of literature-curated PPI in a *S. cerevisiae* network (17)	2016	16 481
FlyNet	Integrated network	13 119	779 484

## NETWORK ASSESSMENT

**Figure 1.**
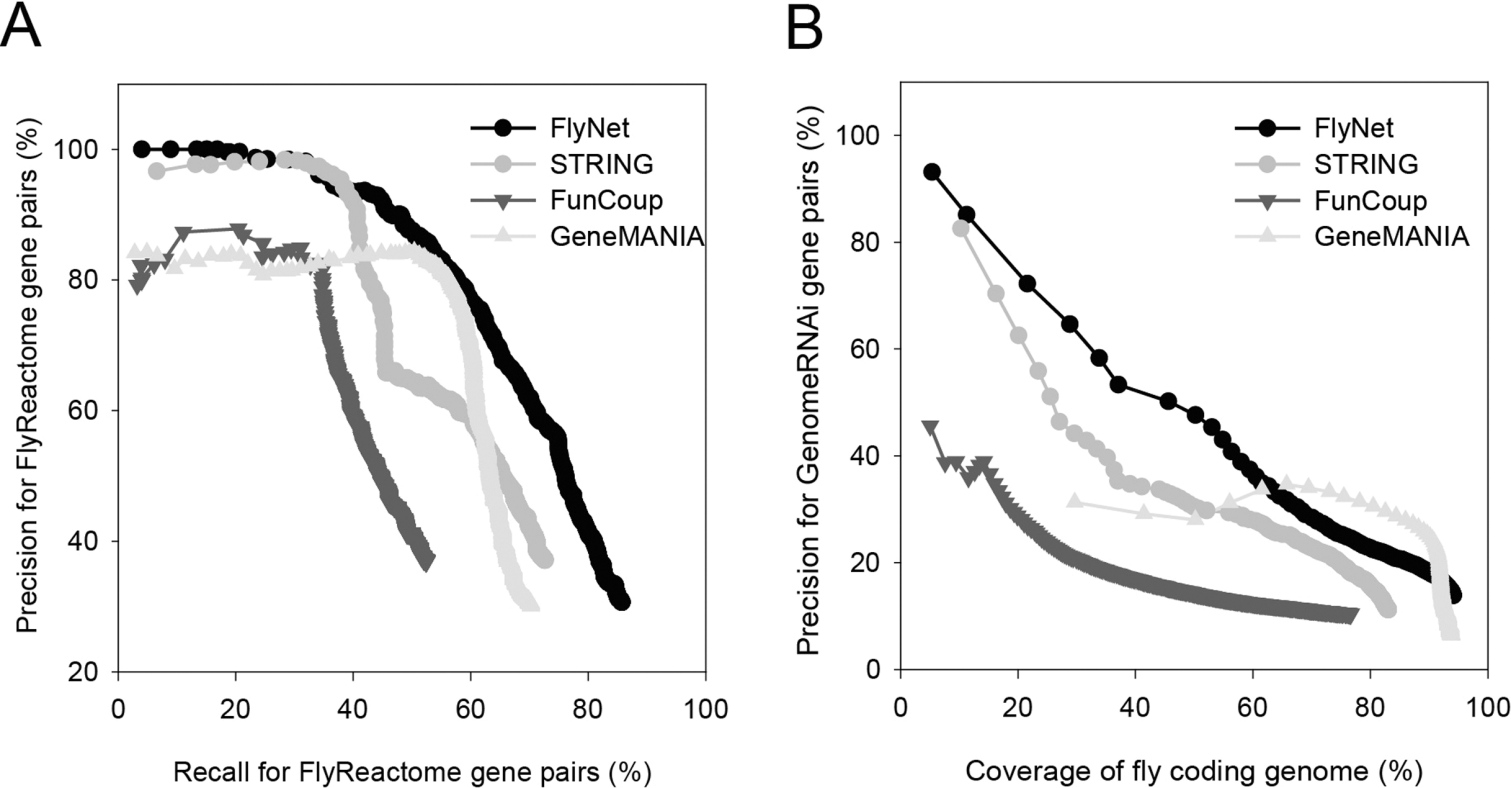
The assessment of FlyNet using gene pairs derived from (**A**) FlyReactome annotations and (**B**) GenomeRNAi phenotype terms. The precision of the gene pairs was measured every 1000 pairs, which were sorted by the network edge weight scores. For the assessment by GenomeRNAi, the coverage of the fly genome was used, because the number of gene pairs by the RNAi phenotype is so large (316 924) that the recall for the majority of the gene pairs is not feasible.

To assess the compatibility of the fly gene networks with fly biological pathways, we employed the FlyReactome database (22), which is independent from the gold-standard data set that was used for the network training. FlyReactome contains only expert-curated *Drosophila* core pathways and reactions. Gene pairs that share pathway terms and those that do not share pathway terms generated 1305 positive and 14 365 negative gene pairs, respectively, for validation. The accuracy of the networks for the given coverage of the positive gene pairs was analyzed by a precision-recall curve. The performance of FlyNet in the retrieval of core pathway links was superior to other fly gene networks from STRING (23), FunCoup (24) and GeneMANIA (25) (Figure 1A).

Because functionally associated genes tend to share loss-of-function phenotypes, we expected that the more accurate networks would include a higher percentage of gene pairs that share RNAi phenotypes. We generated 316 924 positive and 7 107 807 negative gene pairs that share or do not share, respectively, RNAi phenotypes from the GenomeRNAi database (26). Compared with other network servers, FlyNet contained a higher percentage of gene pairs that share RNAi phenotypes for the given genome coverage of the network nodes (Figure 1B). Among the 21 component networks, the highest percentage of gene pairs in FlyNet originated from the co-expression network of fly genes (DM-CX) (Supplementary Figure S1). To determine the possibility of circular reasoning when predicting published RNAi phenotypes, we also assessed FlyNet with the co-citation links (DM-CC) excluded. We found that links derived from the DM-CC have no significant effect on the performance of FlyNet, as assessed by the RNAi phenotype data (Supplementary Figure S2). We measured the overlap between the FlyNet training data and the two validation data sets to confirm their independence. We found that only 33.5% of the FlyReactome and 1.2% of the GenomeRNAi positive gene pairs overlapped with the network training data. Therefore, the higher accuracy of FlyNet compared with other fly gene networks may be attributed, at least in part, to the network construction procedure, which is maximally optimized to the *Drosophila* biology. Taken together, we conclude that FlyNet is a highly accurate and comprehensive functional gene network that can effectively reconstruct both core pathways and phenotypes in *Drosophila*.

## NETWORK PRIORITIZATION OPTIONS

### Distinctive features of the FlyNet web server

In addition to FlyNet, there are currently two other publicly available fly network prioritization servers: GeneMANIA and FunCoup. However, these two servers are not dedicated to *Drosophila*. In addition, compared with these servers, FlyNet has several distinctive features that enable more versatile hypothesis generation for *Drosophila*-based studies: (i) FlyNet can prioritize both genes (*gene prioritization*) and functional annotations (*function prioritization*); (ii) FlyNet can use two complementary network prioritization algorithms, *direct neighborhood* and *network diffusion*, compared with other publicly available network prioritization servers, which provide only one or the other algorithm for use; (iii) FlyNet can utilize a spatiotemporal-specific network (STN) as an additional prioritization strategy for traits associated with a specific developmental stage or tissue and (iv) FlyNet can prioritize *Drosophila* genes not only for *Drosophila* traits (*Fly prioritizer*) but also for human disease (*Human prioritizer*).

### Fly prioritizer–function prioritization

**Figure 2.**
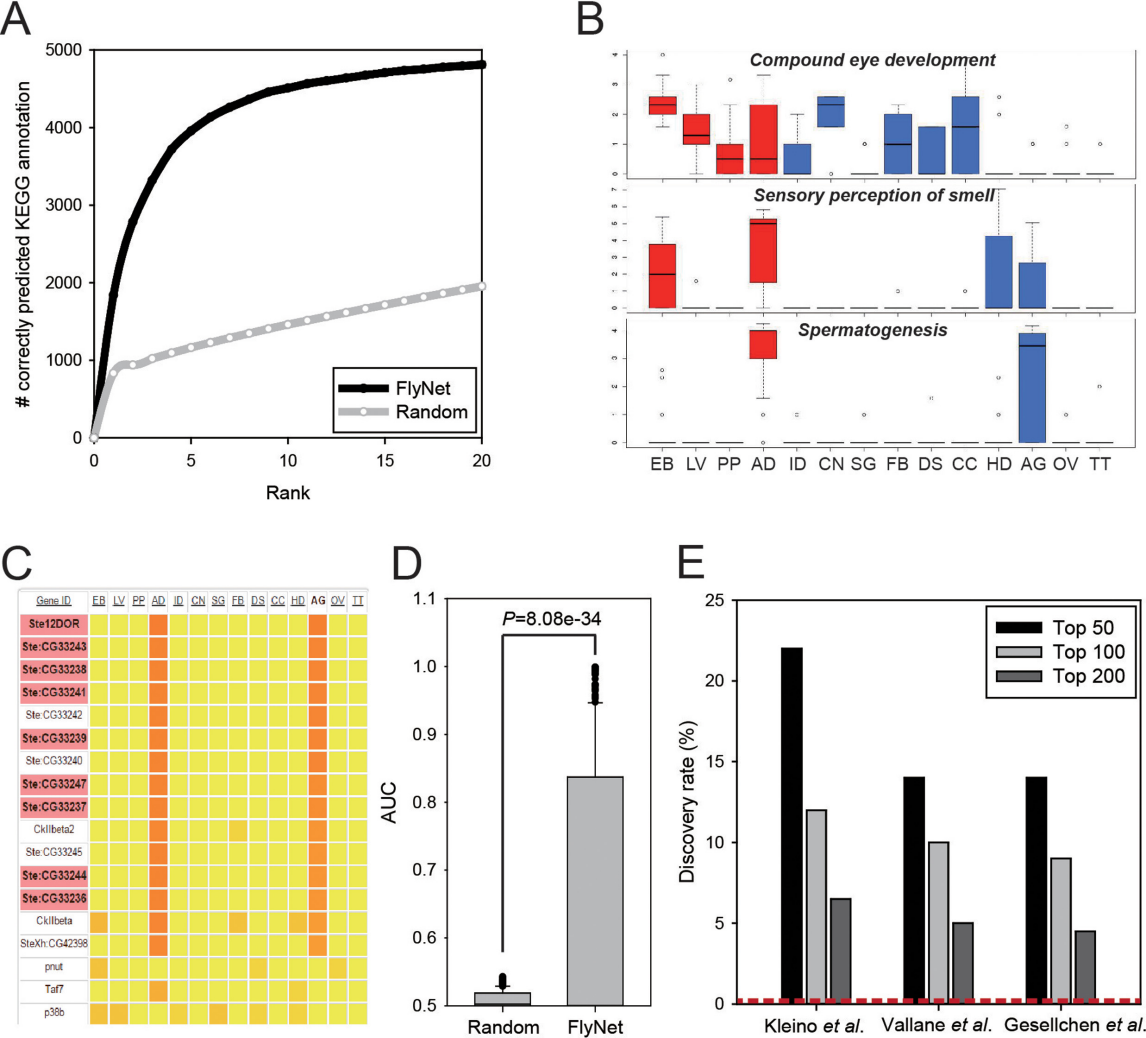
Fly prioritizer analyses. (**A**) The assessment of function prioritization using 5434 KEGG annotations for 2690 fly genes. The number of correctly predicted KEGG terms (y-axis) for the given top rank (x-axis) was assessed for FlyNet and random networks. (**B**) The results of the STN enrichment analysis for three GO-BP terms: compound eye development, sensory perception of smell and spermatogenesis. The logarithm of the number of unique network neighbors for each developmental stage or tissue type was calculated for all guide genes and represented as bar graphs. The codes for the four developmental stages are: EB, embryo; LV, larvae; PP, pupae and AD, adult. The codes for the 10 tissue types are: AG, accessory gland; CC, carcass; CN, central nervous system; DS, digestive system; FB, fat body; HD, head; ID, imaginal disc; OV, ovaries; SG, salivary gland and TT, testes. (**C**) A heat map of the STN scores for spermatogenesis after sorting for the most enriched tissue type, the accessory gland (AG). (**D**) The assessment of gene prioritization for 389 RNAi phenotypes derived from the GenomeRNAi database. (**E**) The discovery rates for the top 50, 100 and 200 novel candidate genes by FlyNet with three independent RNAi screens for the Imd pathway based on the Imd pathway genes annotated by the FlyReactome database. The discovery rate by random chance is ∼1.6%, which is indicated by the red dotted line.

This option prioritizes functional annotations based on GO-BP terms and GenomeRNAi phenotypic terms for a query gene. The functional annotation terms are ranked by the sum of the network edge weight scores (i.e. the sum of the log-likelihood scores in FlyNet) from the query gene to all neighbor genes annotated by each annotation term. We used all 5434 Kyoto Encyclopaedia of Genes and Genomes (KEGG) (27) pathway terms for 2690 fly genes to assess the predictive power of the function prioritization option in FlyNet. The KEGG pathway database was chosen, because the data are well structured and highly curated, and was not used to train FlyNet. We prioritized the KEGG terms for the 2690 genes and counted the number of retrieved terms that were correct among the top-ranked predictions. We found that 4510 (83.0%) KEGG terms were correctly retrieved within the top 10 candidates and 4812 (88.6%) were retrieved within the top 20 candidates, whereas only 1460 (26.9%) and 1953 (35.9%) terms, respectively (Figure 2A), were retrieved by random prediction. These results indicate that FlyNet can effectively predict novel functional annotations for a query gene.

### Fly prioritizer–gene prioritization

This option prioritizes fly genes for a given function, pathway and phenotype that are specified by user-input genes. Because these user-input genes guide the search for new candidates in the network, these genes are called *guide genes*. Upon prioritization, the rank of each fly gene is determined from its association with the guide genes. FlyNet can use two complementary network algorithms to measure the association of each fly gene with the guide gene: *direct neighborhood* and *network diffusion*. Network diffusion algorithms propagate guide gene information throughout the entire network, whereas direct neighborhood algorithms propagate guide gene information only to directly connected neighbours. These algorithms are complementary, and therefore it is advantageous to use both algorithms for prioritization. FlyNet uses the sum of the edge weight scores for the direct neighborhood algorithm and Gaussian smoothing for the network diffusion algorithm, the latter of which is also used in GeneMANIA (25). Based on the prioritized genes, FlyNet first measures the retrieval power for the guide genes to estimate the prediction power of FlyNet for the given traits depicted by these guide genes. The prediction power is assessed by receiver operating characteristic (ROC) analyses and summarized as an area under the ROC curve (AUC) score. The traits with high AUC scores are more likely to benefit from network prioritization.

One distinctive feature of FlyNet is the use of STN as an additional strategy to predict genes for traits associated with a specific developmental stage or tissue. If the known genes for a trait (i.e. guide genes) generally have more network neighbors in a specific developmental stage or tissue type, then novel candidate genes with the same spatiotemporal specificity in network neighbors are more likely to be confident candidates. For this analysis, we first constructed networks for each of the four developmental stages and 10 tissue types in *Drosophila* by filtering the FlyNet edges for genes with a BPKM (i.e. bases per kilobase per million mapped bases) > 1, based on the recent *Drosophila* transcriptome data from the modENCODE project (28). We then compared the networks to identify specific network links for each of four developmental stages. We also compared the networks to identify specific network links for each of 10 tissue types. The resultant 14 STNs are summarized in Supplementary Table S2, and their edge information can be downloaded from the FlyNet server. To measure the functional spatiotemporal specificity for each gene, we counted the number of network neighbors across the 14 STNs. This method provides an opportunity to further select candidate genes based on the STN neighbors. For example, genes annotated by the GO-BP terms ‘compound eye development’, ‘sensory perception of smell’ and ‘spermatogenesis’ exhibit the highest number of STN neighbors for the central nervous system (CN), head (HD) and accessory gland (AG), respectively (Figure 2B). Users also can visualize a heat map that displays the number of STN neighbors across the 14 spatiotemporal contexts for guide genes and candidate genes; an example for spermatogenesis is shown in Figure 2C. If users click the tissue most enriched for STN neighbours (e.g. the AG for spermatogenesis), all the genes are sorted by the number of STN neighbours. As expected, the top-ranked genes are highly enriched for guide genes (highlighted in red), which have many AG-specific network neighbours.

The gene prioritization module also provides several additional analysis results: (i) the visualization of a network of guide genes and a network of a combined set of guide genes and candidate genes using Cytoscape web (29); (ii) the lists of guide genes and candidate genes and (iii) a gene set analysis for guide genes, candidate genes or their combined set with GO-BP, KEGG, BioCyc and GenomeRNAi terms using the hypergeometric test. In particular, we adapted REVIGO (30) to effectively summarize and visualize redundant information in the GO-BP terms.

To assess the predictive power of FlyNet for fly gene prioritization, we performed an ROC analysis for 389 RNAi phenotypes from the GenomeRNAi database (31). We confirmed that the predictive power of FlyNet is significantly higher than random models (*P* = 8.08e-34, Wilcoxon signed-rank test) (Figure 2D). We also assessed the predictive power of FlyNet using multiple guide gene sets for the same pathway derived from independent RNAi screens. We collected three gene sets for the ‘Imd pathway’, which were derived from three independent screens by Gesellchen *et al*. (32) (21 genes), Kleino *et al*. (33) (eight genes) and Valanne *et al*. (34) (30 genes), and submitted each of these three sets for gene prioritization. The validation of the three prediction results by the 22 ‘Imd pathway’ genes annotated in FlyReactome demonstrated discovery rates of 14–22% and 9–12% for the top 50 and 100 candidates, respectively (Figure 2E). These results represent 88–138 fold and 56–75 fold enrichment, respectively, compared with the expected discovery rate by random chance (i.e. 22 Imd pathway genes represent ∼0.16% of the 13 942 genes in the fly genome).

### Human prioritizer–human disease prioritization

*Drosophila* models have been used for the study of neurodevelopmental disorders, cancer and other human diseases (5,35,36). Fly genes and fly mutants are valuable resources in human disease research studies. Therefore, we developed FlyNet to prioritize *Drosophila* genes not only for fly traits (*Fly prioritizer*) but also for human diseases (*Human prioritizer*). The human prioritizer attempts to identify novel human disease genes that can be used to construct a human disease model in the fly, which may allow for genetic screens of disease gene modulators and may identify new therapeutic targets. The human prioritizer option uses the same methods as the fly prioritizer, including the direct neighborhood algorithm, with an additional step for orthology mapping between fly and human genes. Users may submit either fly genes or human genes as guide genes. For example, disease-associated fly genes derived from ‘FlyBase human disease alleles’ or human disease genes from ‘OMIM morbidmap’ (37) can be used as guide genes. These genes are available from the web server, and users can submit these genes with a simple click.

**Figure 3.**
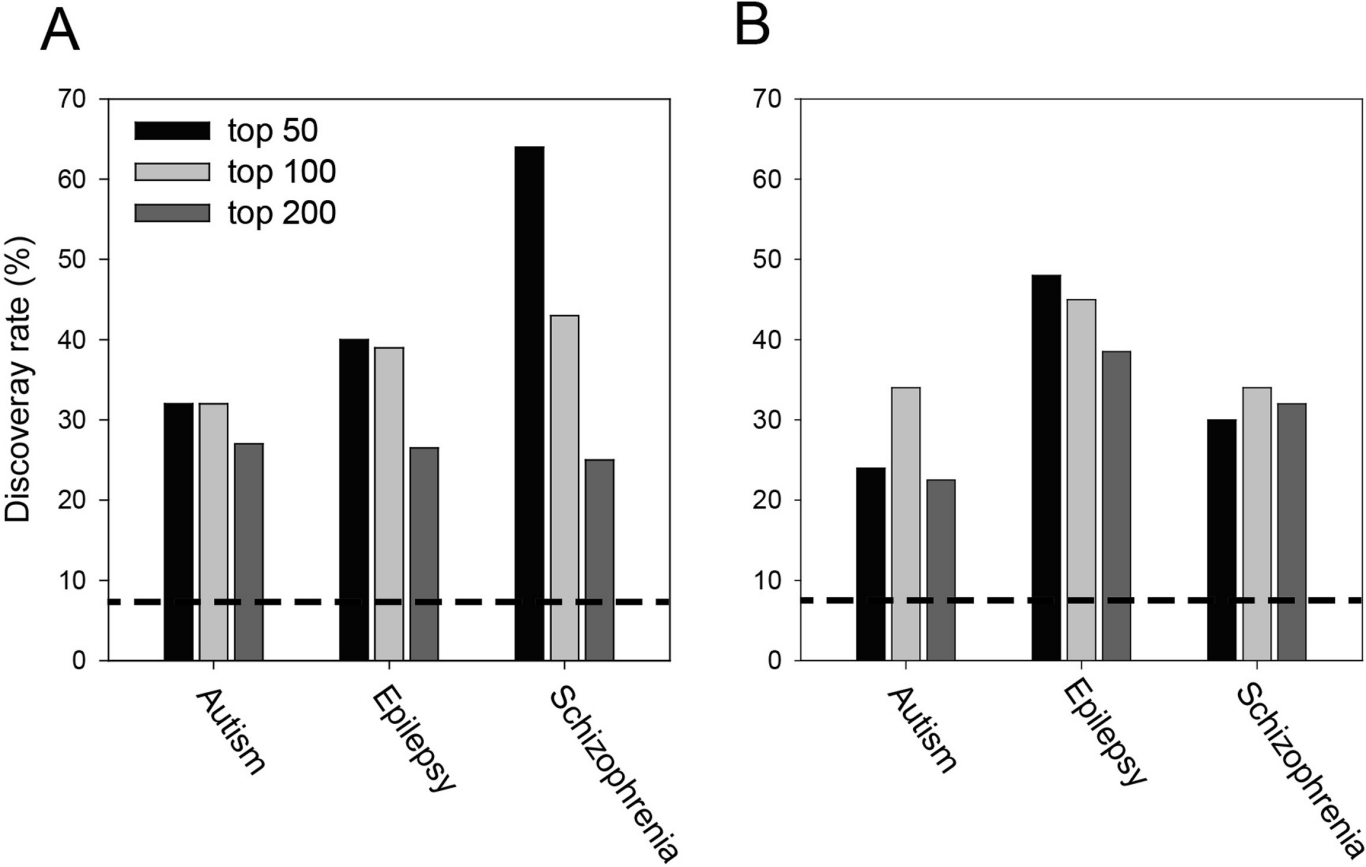
Human prioritizer analyses. Novel candidate genes for autism, epilepsy and schizophrenia were predicted by FlyNet, and top-ranked genes were validated using (**A**) 138 neurological disease genes identified from a recent mutagenesis screen for X-chromosome fly genes and (**B**) 800 genes with *de novo* mutations for autism, epilepsy or schizophrenia. The discovery rates for the top 50, 100 and 200 candidates are represented on the bar graph, and the expected discovery rates by random chance are indicated by the dotted lines.

To assess the predictive power of the human prioritizer for disease genes, we tested various neurodevelopmental disease genes derived from the OMIM database. We submitted human OMIM genes for autism, epilepsy and schizophrenia as guide genes. To validate the resultant novel candidate genes for each neurodevelopmental disease, we used 138 human orthologs of X-chromosome fly genes whose associations with human neurological diseases were reported by a recent mutagenesis screen (5). By random chance, the discovery rate for identifying these 138 genes out of 1710 human orthologs of X-chromosome fly genes is 8.1%. In contrast, we observed a discovery rate of 32–64% for the top 50 candidate genes for the diseases, which represents a 4–8-fold enrichment due to the network prioritization (Figure 3A). We also used *de novo* mutations in autism (480 human genes), epilepsy (204 human genes) or schizophrenia (695 human genes) collected from various studies (see Supplementary Online Methods) to assess the network prioritization for the same three neurodevelopmental disorders. Because there is a significant overlap of genes between these neurological diseases (38), we used the union of these three gene sets (800 genes) to assess the prediction for each disorder. We observed a discovery rate of 34–45% for the top 100 candidate genes for these three disorders, which represents a 4.2–5.6-fold enrichment due to the network prioritization compared with random expectation (i.e. 800 genes represent 8.1% of the 9857 human orthologs in the fly genome) (Figure 3B).

Human genetics studies have revealed that human disease is associated with multiple genes. In particular, thousands of genes associated with heritable disorders have been identified via GWAS and *de novo* mutation screens. We hypothesized that the integration of genetic evidence with network evidence may give rise to more confident candidate genes, which then could be validated experimentally. Therefore, the FlyNet server implements an option to filter novel candidates with network prioritization using *support genes*, which generally are derived from human genetics studies. FlyNet currently provides support genes derived from many human GWAS and *de novo* mutation screens, and these genes can be submitted as support genes with a simple click. Users also can submit their own support genes. If a user chooses this option, then the report page will display two separate lists of candidate genes: (i) *first-tier candidate genes*, which represent the intersection of support genes with the neighbors of guide genes (i.e. the intersection of candidates by human genetic evidence with candidates by network evidence) and (ii) *second-tier candidate genes*, which include all candidates from the network prioritization. The first-tier candidates, which are supported by both network and genetic evidence, are more likely to be promising candidates than those supported by network evidence only. If users need only a handful of candidates with high confidence, then this option may be useful.

## CONCLUSIONS

FlyNet is a freely available network prioritization server dedicated to the *Drosophila* community. A functional gene network of *Drosophila* genes was constructed with machine learning procedures optimized for the *Drosophila* biology. We demonstrated that FlyNet can effectively predict gene functional links, function/pathways, genes for pathways/phenotypes and human disease genes. FlyNet is a distinctive network prioritization server because it prioritizes genes as well as functions, uses two complementary network algorithms and STN and can predict human disease genes. Therefore, FlyNet is a versatile hypothesis-generation server for *Drosophila* biologists who study basic animal genetics as well as human diseases.

## SUPPLEMENTARY DATA

Supplementary Data are available at NAR Online.

SUPPLEMENTARY DATA
