# Supplementary material for: FlyNet: a versatile network prioritization server for the Drosophila community
Source: Nucleic Acids Res. 2015 May 5;43(Web Server issue):W91–7. doi: 10.1093/nar/gkv453 (PMC4489278; doi:10.1093/nar/gkv453)
Supplement: SUPPLEMENTARY DATA [file supp_43_W1_W91__index.html]

FlyNet: a versatile network prioritization server for the Drosophila community — FlyNet: a versatile network prioritization server for the Drosophila community — SUPPLEMENTARY DATA 

# FlyNet: a versatile network prioritization server for the *Drosophila* community

## SUPPLEMENTARY DATA

**Files in this Data Supplement:**

- SUPPLEMENTARY DATA
