# Supplementary material for: FlyNet: a versatile network prioritization server for the Drosophila community
Source: Nucleic Acids Res. 2015 May 5;43(Web Server issue):W91–7. doi: 10.1093/nar/gkv453 (PMC4489278; doi:10.1093/nar/gkv453)
Supplement: SUPPLEMENTARY DATA [file supp_gkv453_nar-00544-web-b-2015-File005.pdf]

Supplementary Online Methods

FlyNet: a versatile network prioritization server for the *Drosophila* community

Junha Shin, Sunmo Yang, Eiru Kim, Chan Yeong Kim, Hongseok Shim, Ara Cho, Hyojin Kim, Sohyun Hwang, Jung Eun Shim and Insuk Lee

Construction of FlyNet

Gold standard set for machine learning

The inferred network links were benchmarked by 'gold standard' pairs of genes annotated by the same gene ontology (1) biological process (GO-BP) terms or MetaCyc (2) terms. GO-BP annotations for *Drosophila melanogaster* genes were based on FlyBase (3) release 5.54. Annotations only supported by IDA (inferred from direct assay), IPI (inferred from protein interaction), ISS (inferred from sequence or structural similarity) and TAS (traceable author statement) were used to achieve the high accuracy of the gold standard data. GO-BP terms below level 11 of the annotation hierarchy were excluded, because they provide highly specific definitions. In addition, large GO-BP terms that generate too many gene pairs, such as 'regulation of transcription, DNA-templated' (GO:0006355) and 'translation' (GO:0006412), 'protein phosphorylation (GO:0006468)', and 'proteolysis (GO:0006508)' were excluded in order to construct a functionally unbiased gold standard data set (4). The resultant gold standard data set from GO-BP terms contains 87,856 positive and 7,778,705 negative pairs between 3,957 genes, covering ~28% of *D. melanogaster* coding genome. MetaCyc annotations were based on BioCyc *Drosophila* annotation version 4.0.1.1.1. Annotations by 18 super-pathways were excluded, because they are broad concepts, in which multiple subordinate pathway concepts are included. The resultant gold standard data set from MetaCyc terms contains 10,526 positive and 296,410 negative pairs between 784 genes. Finally, we combined these two sets to generate the final gold standard set of 97,267 positive and 9,890,948 negative pairs between 4,470 genes, which covers ~32% of *D. melanogaster* genome.

Log likelihood score (LLS) scheme to unify multiple types of data-intrinsic scores

Log likelihood score (LLS), a unified scoring scheme for heterogeneous data types, was previously developed and has proven useful in benchmarking and integrating diverse types of data (5). LLS can be calculated using the following equation:

$$LLS = \ln\left(\frac{P(L|E)/P(\neg L|E)}{P(L)/P(\neg L)}\right)$$

, where $P(L|E)$ and $P(\neg L|E)$ represent the frequencies of positive (L) and negative ($\neg L$) gold standard pathway links observed in the given experimental or computational evidences (E), while $P(L)$ and $P(\neg L)$ represent the prior expectations (i.e., the total frequencies of all positive and negative gold standard pathway gene pairs, respectively). To avoid over-fitting,

we used '0.632 bootstrapping' for all *LLS* calculations because of its credibility in estimating classifier error rates.

For the gene pairs sorted by data-intrinsic scores, *LLS* scores were calculated for bins of equal numbers of gene pairs. A regression between data-intrinsic scores (e.g. mutual information, correlation coefficient and probability) and log likelihood scores based on gold standard gene pairs is used for interpolation to estimate *LLS* of individual gene pairs. Linear fits in general over-estimate *LLS* for the gene pairs in the most significant score range, whereas sigmoidal curve fits result in more conservative *LLS* for the same score range (4).

Weighted Sum (WS) method for network integration

Weighted sum (*WS*) (5,6) is a variant of the naïve Bayesian method, which accounts for the average correlation among integrated data. *WS* is calculated using the following equation:

$$WS = S_0 + \sum_{i=1}^{n} \frac{S_i}{D \cdot i}, for\ all\ S \geq T$$

, where S_0 is the best *LLS* score among all of the available *LLSs* for each link; *D* is a free parameter representing the degree of correlation among the scores; *T* is a threshold of *LLS* to be integrated; and *i* is the rank index from ordering in descending magnitude the *n LLSs* for each link. The values for the free parameters, *D* and *T*, were chosen to maximize overall performance on the benchmarks. To take the best case of integration, we also tested the performance of the naïve Bayesian integration of *LLS* scores, and then selected the integration conditions that maximizes the area under the plot of *LLS* versus genome coverage of the integrated network (4).

Protein-protein interactions – based on high-throughput experiments (HT) and literature curation (LC)

Protein-protein interaction (PPI) data was dealt as two categories: i) PPI by high-throughput experiments such as yeast-two-hybrid assay (Y2H) or affinity purification/mass spectrometry (AP/MS), and ii) PPI by small-scale experiments. Both categories of PPI data were obtained from iRefWeb (7) version 13.0, a consolidated database of several public protein interaction databases. Protein interactions are prioritized based on the significance calculated using Fisher's exact test.

Co-expression (CX) of genes across biological conditions

More than 2,000 microarray and RNA-seq samples are publicly available from Gene Expression Omnibus (GEO) (8). We analyzed GEO series (GSE) based on two Affymetrix chip platforms, GPL72 and GPL1322, which support the largest number of gene expression

samples. GSE with less than 12 samples were excluded, because correlation coefficient by low dimensional data tends to give many promiscuous co-functional links. Overall, 53 GSE comprising 1,873 expression samples were analyzed and the full list of GSE series are represented in **Supplementary Table S1**. The degree of co-expression was measured by Pearson's correlation coefficient.

Comparative genomics-based computational methods – Phylogenetic profiling (PG) and Gene neighborhood (GN)

The phylogenetic profiling (PG) method is based on the observation that functionally related genes tend to be gained or lost together during the evolutionary process (i.e., co-inherited) because they both might be required to operate the same biological pathways. To identify co-functionality between genes from the co-inheritance pattern, we conducted BLASTp for all *D. melanogaster* genes against the genome set generated based on each of three domains of life; sets of 122 completely sequenced genomes for Archaea, 1,626 for Bacteria and 396 for Eukarya. We found that this divide-and-integrate approach based on domain-specific phylogenetic profiles substantially improves network coverage as well as accuracy. Phylogenetic profile matrices were constructed with BLAST hit scores and the similarity between profiles of two genes was calculated as mutual information (*MI*) score as described in a previous study (9).

The gene neighborhood (GN) method is based on the observation that genes located in the bacterial genomic vicinity tend to be co-regulated, and thus functionally associated. We used 1,748 bacterial genomes (122 from Archaea and 1,626 from Bacteria) for the analysis. There are two different measures of genomic vicinity: i) physical distance between neighboring genes (10-12), and ii) relative distance measured by the probability of neighborhood (13). It has been reported that these two methods are complementary and the integration of two methods improves prediction performance of the network (14).

Text mining from research articles – Co-citation (CC)

Co-citation of genes in the same articles is a relatively simple but effective method to identify functionally associated genes (15). We inferred co-citation-based links by scanning PubMed Central full text articles and Medline abstracts (as of January 2014) that contain the word "*melanogaster*". Co-cited genes in the same articles were paired to generate links and measured the statistical significance using Fisher's exact test.

Co-occurrence of protein domains – Domain co-occurrence (DC)

Because protein domains are generally considered as functional units, proteins that share domains could have similar functions. Based on the presence of domains by InterPro database (16), we generated domain profiles for proteins. With these profiles, we measured

significance of domain co-occurrence between two proteins. Accounting for inverse relationship between occurrence and functional specificity of domains, we used ‘weighted mutual information’ scheme which gives more weight on rarer domains.

Functional information transferred by orthology – Associalogs

Due to the evolutionary conservation of biological pathways across species, functional association between genes in a target organism can be inferred from a functional association between orthologs in a reference organism, based on the algorithm namely ‘associalog’ (6). We used Inparanoid (17) for the orthology mapping, which allows identification of co-orthologs. We transferred co-functional links from AraNet v2 (18), WormNet v3 (19), YeastNet v3 (20), and unpublished co-functional networks for human and zebra fish.

Spatiotemporal-specific network (STN)

The data of spatiotemporal expressions of *D. melanogaster* genes were obtained from the recent *Drosophila* transcriptome atlas data from the modENCODE project (21). For all expression samples based on RNA-seq, we took genes with BPKM (bases per kilobase per million mapped bases) > 1 only. We classified 41 selected spatiotemporal expression samples into four developmental stages (embryo, larvae, pupae and adult) and ten tissue types (imaginal disc, CNS, salivary gland, fat body, digestive system, carcass, heads, accessory gland, ovary and testes). As the result, we generated 14 sets of genes associated with different developmental stages and tissue types. These 14 gene sets were used to filter FlyNet for 14 spatiotemporal networks. We then compared the networks for four developmental stages to identify specific network links for each developmental stage, and compared the networks for ten tissue types to identify specific network links for each tissue type. These *spatiotemporal-specific links* (i.e., links found in only one of four developmental stages or in only one of ten tissue types) generate 14 spatiotemporal-specific networks (STNs) summarized in **Supplementary Table S2**. Edge information of the 14 STNs are also available from FlyNet server.

Dataset for the assessment of human disease prioritization

Fly X-chromosome genes whose human orthologs are associated with neurodevelopmental diseases were collected from a recent study of fly mutagenesis screen (22). Human genes with *de novo* mutations in neurological disorders from the following references: autism (23-26), epilepsy (27), and schizophrenia (28-31).

Supplementary Figure 1. The assessment of 21 component networks of the FlyNet against gene pairs derived from GenomeRNAi phenotype terms

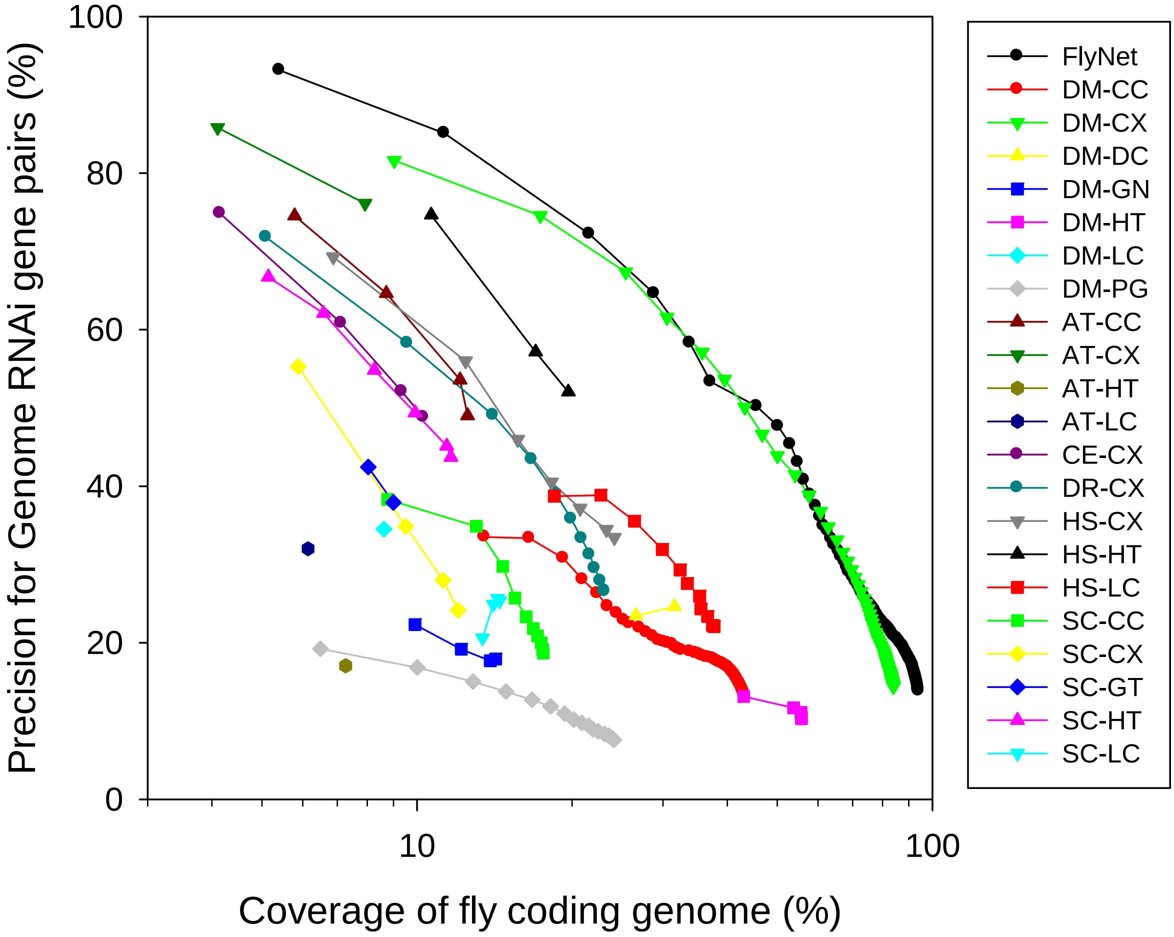

Supplementary Figure 2. The assessment of networks including FlyNet with no links by co-cited fly genes (FlyNet w/o DM-CC) using gene pairs derived from GenomeRNAi phenotype terms.

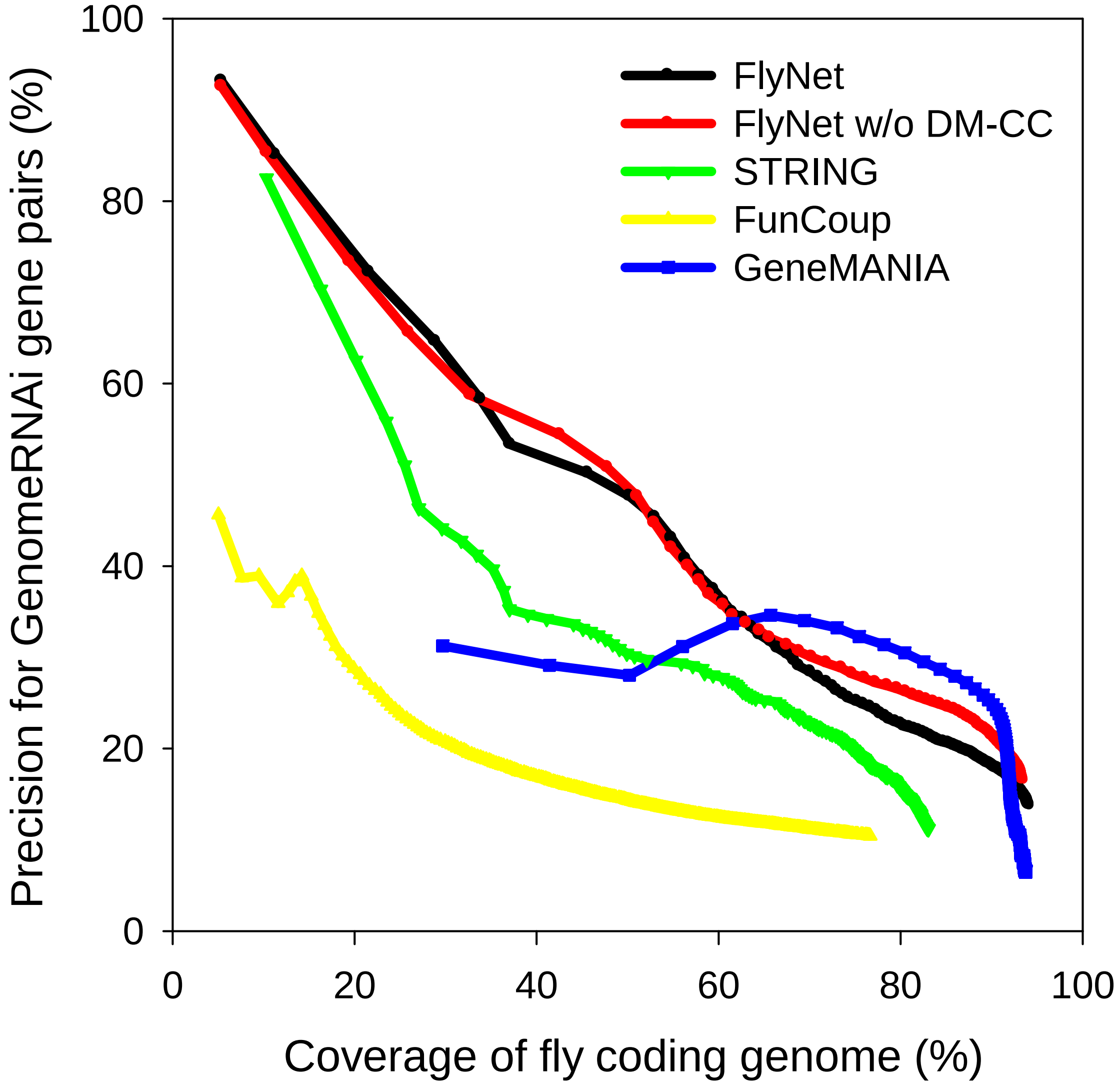

Supplementary Table S1. Summary of FlyNet construction

	Description	# Genes	# Links
Network genes	13,942 protein coding genes from FlyBase r5.54		
Gold standard gene pairs for training	Gene pairs that share the same Gene Ontology biological process terms as supported by GO evidence of IDA, IPI, ISS, TAS, or same MetaCyc pathway terms	4,470	97,267
Network size	779,484 links, 13,119 genes (94.1% of FlyBase r5.54 coding genes)	13,119	779,484
DM-CC	Co-citation of genes in PubMed Central full text articles and Medline abstracts	6,027	503,475
DM-CX	Integrated co-expression gene links from 53 GEO series comprising 1,873 experiments using the Affymetrix DNA-chip (GPL : GSEs) (GPL72 : GSE3057, 3060, 3854, 6493, 6558, 7159, 8751, 9889, 10012, 11203, 42255) (GPL1322 : GSE2863, 5404, 5430, 7614, 7763, 8775, 8892, 8938, 9107, 10940, 11695, 12160, 14517, 14531, 14779, 16152, 16713, 17013, 17803, 17874, 21520, 22308, 23802, 24729, 24917, 24978, 26246, 26726, 27163, 27345, 31564, 33100, 33779, 33801, 34400, 36582, 37708, 38036, 46550, 47176, 48385, 48997)	11,718	275,033
DM-DC	Co-occurrence of InterPro protein domains between two genes	4,407	7,604
DM-GN	Genomic proximity, integration of a distance-based (DGN) and a probability-based (PGN)	1,979	15,820
DM-HT	PPI from high-throughput experiments, downloaded from iRefWeb v13.0	7,759	15,820
DM-LC	PPI excluding high-throughput experiments, downloaded from iRefWeb v13.0	1,202	2,226
DM-PG	Integrated links of 3 networks from phylogenetic profiles of 122 Archaea genomes, 1626 bacterial genomes and 396 Eukaryote genomes	3,357	80,506
AT-CC	Associalogs from AraNet v2	1,747	17,502
AT-CX	Associalogs from AraNet v2, integrated co-expression gene links from 18 GEO series (GSE1491, 4847, 5629-34, 5636-39, 9674, 10670, 11262, 12402, 12403, 13739, 18071, 18971, 18975, 18978, 19700, 21684, 30492, 35325, 35544)	1,105	9,455
AT-HT	Associalogs from AraNet v2, PPI from 4 high-throughput screens	1,013	2,823
AT-LC	Associalogs from AraNet v2, PPI from TAIR, IntAct, MINT, BioGrid, DIP	856	1,977
CE-CX	Associalogs from WormNet v3, integrated co-expression gene links from 2 GEO series (GSE2180, 11055)	1,434	17,497
DR-CX	Integrated associalogs of co-expression gene links from 8 GEO series (GSE9020, 10188, 11893, 11107, 14495, 16264, 39731, 48806)	3,223	55,515
HS-CX	Integrated associalogs of co-expression gene links from 8 GEO series (GSE3307, 7390, 9419, 9874, 11877, 11903, 14994, 37201)	3,366	32,482
HS-HT	Integrated associalogs of high-throughput physical interactions from 6 literature sources	2,741	12,520
HS-LC	Integrated associalogs from the PPI network from HPRD, BioGrid, IntAct, MINT, DIP, iRefWeb	5,254	50,488
SC-CC	Associalogs from YeastNet v3	2,449	48,473
SC-CX	Integrated associalogs of co-expression gene links from 7 GEO series (GSE12442, 16799, 17716, 24888, 25582, 36954, 38848)	1,674	18,488

SC-GT	Associalogs from YeastNet v3	1,254	6,482
SC-HT	Associalogs from YeastNet v3	1,622	18,300
SC-LC	Associalogs from YeastNet v3	2,016	16,481

Each datasets are denoted as XX-YY, where XX represents species names (DM: *Drosophila melanogaster*, AT: *Arabidopsis thaliana*, CE: *Caenorhabtitis elegans*, DR: *Danio rerio*, HS: *Homo sapiens*, SC: *Saccharomyces cerevisiae*), and YY represents the data type (CX: inferred from co-expression of genes, CC: inferred from co-citation, DC: inferred from domain co-occurrence, GN: inferred from gene neighborhood, GT: inferred from genetic interaction, HT: inferred from high-throughput protein-protein interactions, LC: inferred from literature curated protein-protein interactions, PG: inferred from phylogenetic profile similarity)

Supplementary Table S2. Summary of spatiotemporal-specific network (STN)

Specific networks		Used expression set	# Genes	# Links
Developmental Stage-specific	Embryo	em0-2hr<br>em2-4hr<br>em4-6hr<br>em6-8hr<br>em8-10hr<br>em10-12hr<br>em12-14hr<br>em14-16hr<br>em16-18hr<br>em18-20hr<br>em20-22hr<br>em22-24hr	3,183	7,169
	Larvae	L3_Carcass<br>L3_CNS<br>L3_DigestiveSystem<br>L3_FatBody<br>L3_ImaginalDiscs<br>L3_SalivaryGlands	1,316	2,135
	Pupae	WPP_2days_CNS<br>WPP_2days_Fat<br>WPP_FatBody<br>WPP_SalivaryGlands	1,250	1,892
	Adult	AdMatedF_Ecl_1day_Heads<br>AdMatedF_Ecl_4days_Heads<br>AdMatedF_Ecl_20days_Heads<br>AdVirginF_Ecl_1day_Heads<br>AdVirginF_Ecl_4days_Heads<br>AdVirginF_Ecl_20days_Heads<br>AdMatedM_Ecl_1day_Heads<br>AdMatedM_Ecl_4days_Heads<br>AdMatedM_Ecl_20days_Heads<br>AdMixedMF_Ecl_1day_Carcass<br>AdMixedMF_Ecl_4days_Carcass<br>AdMixedMF_Ecl_20days_Carcass<br>AdMixedMF_Ecl_1day_DigestiveSystem<br>AdMixedMF_Ecl_4day_DigestiveSystem<br>AdMixedMF_Ecl_20days_DigestiveSystem<br>AdMatedM_Ecl_4days_Testes<br>AdMatedM_Ecl_4days_AccessoryGlands<br>AdVirginF_Ecl_4days_Ovaries<br>AdMatedF_Ecl_4days_Ovaries	4,766	14,445

Tissue-specific	Imaginal Disc	L3_ImaginalDiscs	1,004	1,160
	Central Nervous System	L3_CNS<br>WPP_2days_CNS	1,108	1,613
	Salivary Gland	L3_SalivaryGlands<br>WPP_SalivaryGlands	667	805
	Fat Body	L3_FatBody<br>WPP_2days_Fat<br>WPP_FatBody	2,051	3,314
	Digestive System	AdMixedMF_Ecl_1day_DigestiveSystem<br>AdMixedMF_Ecl_4day_DigestiveSystem<br>AdMixedMF_Ecl_20days_DigestiveSystem<br>L3_DigestiveSystem	1,981	3,758
	Carcass	AdMixedMF_Ecl_1day_Carcass<br>AdMixedMF_Ecl_4days_Carcass<br>AdMixedMF_Ecl_20days_Carcass<br>L3_Carcass	1,537	2,421
	Heads	AdMatedF_Ecl_1day_Heads<br>AdMatedF_Ecl_4days_Heads<br>AdMatedF_Ecl_20days_Heads<br>AdVirginF_Ecl_1day_Heads<br>AdVirginF_Ecl_4days_Heads<br>AdVirginF_Ecl_20days_Heads<br>AdMatedM_Ecl_1day_Heads<br>AdMatedM_Ecl_4days_Heads<br>AdMatedM_Ecl_20days_Heads	2,062	3,741
	Accessory Gland	AdMatedM_Ecl_4days_AccessoryGlands	556	632
	Ovaries	AdVirginF_Ecl_4days_Ovaries<br>AdMatedF_Ecl_4days_Ovaries	402	455
	Testes	AdMatedM_Ecl_4days_Testes	1,010	1,036

Supplementary references

1. Ashburner, M., Ball, C.A., Blake, J.A., Botstein, D., Butler, H., Cherry, J.M., Davis, A.P., Dolinski, K., Dwight, S.S., Eppig, J.T. *et al.* (2000) Gene ontology: tool for the unification of biology. The Gene Ontology Consortium. *Nature genetics*, **25**, 25-29.
2. Caspi, R., Altman, T., Dreher, K., Fulcher, C.A., Subhraveti, P., Keseler, I.M., Kothari, A., Krummenacker, M., Latendresse, M., Mueller, L.A. *et al.* (2012) The MetaCyc database of metabolic pathways and enzymes and the BioCyc collection of pathway/genome databases. *Nucleic acids research*, **40**, D742-753.
3. St Pierre, S.E., Ponting, L., Stefancsik, R., McQuilton, P. and FlyBase, C. (2014) FlyBase 102--advanced approaches to interrogating FlyBase. *Nucleic Acids Res*, **42**, D780-788.
4. Lee, I., Li, Z. and Marcotte, E.M. (2007) An improved, bias-reduced probabilistic functional gene network of baker's yeast, Saccharomyces cerevisiae. *PloS one*, **2**, e988.
5. Lee, I., Date, S.V., Adai, A.T. and Marcotte, E.M. (2004) A probabilistic functional network of yeast genes. *Science*, **306**, 1555-1558.
6. Lee, I., Lehner, B., Crombie, C., Wong, W., Fraser, A.G. and Marcotte, E.M. (2008) A single gene network accurately predicts phenotypic effects of gene perturbation in Caenorhabditis elegans. *Nature genetics*, **40**, 181-188.
7. Turner, B., Razick, S., Turinsky, A.L., Vlasblom, J., Crowdy, E.K., Cho, E., Morrison, K., Donaldson, I.M. and Wodak, S.J. (2010) iRefWeb: interactive analysis of consolidated protein interaction data and their supporting evidence. *Database (Oxford)*, **2010**, baq023.
8. Barrett, T., Troup, D.B., Wilhite, S.E., Ledoux, P., Rudnev, D., Evangelista, C., Kim, I.F., Soboleva, A., Tomashevsky, M., Marshall, K.A. *et al.* (2009) NCBI GEO: archive for high-throughput functional genomic data. *Nucleic acids research*, **37**, D885-890.
9. Date, S.V. and Marcotte, E.M. (2003) Discovery of uncharacterized cellular systems by genome-wide analysis of functional linkages. *Nat Biotechnol*, **21**, 1055-1062.
10. Dandekar, T., Snel, B., Huynen, M. and Bork, P. (1998) Conservation of gene order: a fingerprint of proteins that physically interact. *Trends Biochem Sci*, **23**, 324-328.
11. Overbeek, R., Fonstein, M., D'Souza, M., Pusch, G.D. and Maltsev, N. (1999) The use of gene clusters to infer functional coupling. *Proc Natl Acad Sci U S A*, **96**, 2896-2901.
12. Korbel, J.O., Jensen, L.J., von Mering, C. and Bork, P. (2004) Analysis of genomic context: prediction of functional associations from conserved bidirectionally transcribed gene pairs. *Nat Biotechnol*, **22**, 911-917.
13. Bowers, P.M., Pellegrini, M., Thompson, M.J., Fierro, J., Yeates, T.O. and Eisenberg, D. (2004) Prolinks: a database of protein functional linkages derived from coevolution. *Genome Biol*, **5**, R35.
14. Shin, J., Lee, T., Kim, H. and Lee, I. (2014) Complementarity between distance- and probability-based methods of gene neighbourhood identification for pathway reconstruction. *Mol Biosyst*, **10**, 24-29.
15. Jenssen, T.K., Laegreid, A., Komorowski, J. and Hovig, E. (2001) A literature network of human genes for high-throughput analysis of gene expression. *Nat Genet*, **28**, 21-28.
16. Hunter, S., Jones, P., Mitchell, A., Apweiler, R., Attwood, T.K., Bateman, A., Bernard, T., Binns, D., Bork, P., Burge, S. *et al.* (2012) InterPro in 2011: new developments in

the family and domain prediction database. *Nucleic acids research*, **40**, D306-312.
17. Ostlund, G., Schmitt, T., Forslund, K., Kostler, T., Messina, D.N., Roopra, S., Frings, O. and Sonnhammer, E.L. (2010) InParanoid 7: new algorithms and tools for eukaryotic orthology analysis. *Nucleic Acids Res*, **38**, D196-203.
18. Lee, T., Yang, S., Kim, E., Ko, Y., Hwang, S., Shin, J., Shim, J.E., Shim, H., Kim, H., Kim, C. *et al.* (2015) AraNet v2: an improved database of co-functional gene networks for the study of Arabidopsis thaliana and 27 other nonmodel plant species. *Nucleic Acids Res*, **43**, D996-D1002.
19. Cho, A., Shin, J., Hwang, S., Kim, C., Shim, H., Kim, H., Kim, H. and Lee, I. (2014) WormNet v3: a network-assisted hypothesis-generating server for Caenorhabditis elegans. *Nucleic Acids Res*, **42**, W76-82.
20. Kim, H., Shin, J., Kim, E., Kim, H., Hwang, S., Shim, J.E. and Lee, I. (2014) YeastNet v3: a public database of data-specific and integrated functional gene networks for Saccharomyces cerevisiae. *Nucleic Acids Res*, **42**, D731-736.
21. Brown, J.B., Boley, N., Eisman, R., May, G.E., Stoiber, M.H., Duff, M.O., Booth, B.W., Wen, J., Park, S., Suzuki, A.M. *et al.* (2014) Diversity and dynamics of the Drosophila transcriptome. *Nature*, **512**, 393-399.
22. Yamamoto, S., Jaiswal, M., Charng, W.L., Gambin, T., Karaca, E., Mirzaa, G., Wiszniewski, W., Sandoval, H., Haelterman, N.A., Xiong, B. *et al.* (2014) A drosophila genetic resource of mutants to study mechanisms underlying human genetic diseases. *Cell*, **159**, 200-214.
23. Sanders, S.J., Murtha, M.T., Gupta, A.R., Murdoch, J.D., Raubeson, M.J., Willsey, A.J., Ercan-Sencicek, A.G., DiLullo, N.M., Parikshak, N.N., Stein, J.L. *et al.* (2012) De novo mutations revealed by whole-exome sequencing are strongly associated with autism. *Nature*, **485**, 237-241.
24. O'Roak, B.J., Vives, L., Girirajan, S., Karakoc, E., Krumm, N., Coe, B.P., Levy, R., Ko, A., Lee, C., Smith, J.D. *et al.* (2012) Sporadic autism exomes reveal a highly interconnected protein network of de novo mutations. *Nature*, **485**, 246-250.
25. Neale, B.M., Kou, Y., Liu, L., Ma'ayan, A., Samocha, K.E., Sabo, A., Lin, C.F., Stevens, C., Wang, L.S., Makarov, V. *et al.* (2012) Patterns and rates of exonic de novo mutations in autism spectrum disorders. *Nature*, **485**, 242-245.
26. Iossifov, I., Ronemus, M., Levy, D., Wang, Z., Hakker, I., Rosenbaum, J., Yamrom, B., Lee, Y.H., Narzisi, G., Leotta, A. *et al.* (2012) De novo gene disruptions in children on the autistic spectrum. *Neuron*, **74**, 285-299.
27. Epi, K.C., Epilepsy Phenome/Genome, P., Allen, A.S., Berkovic, S.F., Cossette, P., Delanty, N., Dlugos, D., Eichler, E.E., Epstein, M.P., Glauser, T. *et al.* (2013) De novo mutations in epileptic encephalopathies. *Nature*, **501**, 217-221.
28. Girard, S.L., Gauthier, J., Noreau, A., Xiong, L., Zhou, S., Jouan, L., Dionne-Laporte, A., Spiegelman, D., Henrion, E., Diallo, O. *et al.* (2011) Increased exonic de novo mutation rate in individuals with schizophrenia. *Nat Genet*, **43**, 860-863.
29. Xu, B., Ionita-Laza, I., Roos, J.L., Boone, B., Woodrick, S., Sun, Y., Levy, S., Gogos, J.A. and Karayiorgou, M. (2012) De novo gene mutations highlight patterns of genetic and neural complexity in schizophrenia. *Nat Genet*, **44**, 1365-1369.
30. Gulsuner, S., Walsh, T., Watts, A.C., Lee, M.K., Thornton, A.M., Casadei, S., Rippey, C., Shahin, H., Consortium on the Genetics of, S., Group, P.S. *et al.* (2013) Spatial and temporal mapping of de novo mutations in schizophrenia to a fetal prefrontal cortical network. *Cell*, **154**, 518-529.

31. Fromer, M., Pocklington, A.J., Kavanagh, D.H., Williams, H.J., Dwyer, S., Gormley, P., Georgieva, L., Rees, E., Palta, P., Ruderfer, D.M. *et al.* (2014) De novo mutations in schizophrenia implicate synaptic networks. *Nature*, **506**, 179-184.
